# Utility of HR-pQCT in detecting training-induced changes in healthy adult bone morphology and microstructure

**DOI:** 10.3389/fphys.2023.1266292

**Published:** 2023-10-20

**Authors:** Nicole M. Sekel, Julie M. Hughes, Adam J. Sterczala, Kelly H. Mroz, Mita Lovalekar, Jane Cauley, Julie P. Greeves, Bradley C. Nindl

**Affiliations:** ^1^ Neuromuscular Research Laboratory, Warrior Human Performance Research Center, University of Pittsburgh, Pittsburgh, PA, United States; ^2^ Military Performance Division, United States Army Research Institute of Environmental Medicine, Natick, MA, United States; ^3^ Department of Epidemiology, School of Public Health, University of Pittsburgh, Pittsburgh, PA, United States; ^4^ Army Health and Performance Research, UK Army, Andover, United Kingdom

**Keywords:** high-resolution pQCT, X-ray, imaging, exercise training, short-term changes

## Abstract

Healthy bone adjusts its traits in an exceptionally coordinated, compensatory process. Recent advancements in skeletal imaging via High-Resolution Peripheral Quantitative Computed Tomography (HR-pQCT) allows for the *in vivo* 3-dimensional and longitudinal quantification of bone density, microarchitecture, geometry, and parameters of mechanical strength in response to varying strain stimuli including those resulting from exercise or military training. Further, the voxel size of 61 microns has the potential to capture subtle changes in human bone in as little as 8 weeks. Given the typical time course of bone remodeling, short-term detection of skeletal changes in bone microstructure and morphology is indicative of adaptive bone formation, the deposition of new bone formation, uncoupled from prior resorption, that can occur at mechanistically advantageous regions. This review aims to synthesize existing training-induced HR-pQCT data in three distinct populations of healthy adults excluding disease states, pharmacological intervention and nutritional supplementation. Those included are: 1) military basic or officer training 2) general population and 3) non-osteoporotic aging. This review aims to further identify similarities and contrasts with prior modalities and cumulatively interpret results within the scope of bone functional adaptation.

## 1 Introduction

Julius Wolff’s “Law of Bone Transformation” in 1892 and Harold Frost’s “Mechanostat Theory” nearly a century later, laid the foundational framework in which bone—once thought of as an inert, unchanging tissue—functionally adapts to its environment simultaneously meeting the contradictory needs of stiffness and flexibility during impact loading, muscle contraction and joint movement (Wolff, 1986; Frost, 1987; Seeman, 2002; Frost, 2003; Jepsen, 2009). Mechanical loading as a consequence of exercise training causes varying degrees of tissue-level deformation of bone’s extracellular matrix dependent on whole bone stiffness. This deformation results in a strain stimulus which if greater than customary, and heavily influenced by osteocyte viability, may elicit one of two adaptive processes: formation modeling or targeted remodeling (Hughes and Bouxsein, 2020). Targeted remodeling, whereby osteoblast and osteoclast activity are temporally coupled with the objective of resorbing and replacing small packets of damaged tissue is important for modulating microdamage—that is, tissue deterioration at the micro- and nanostructure level (Morgan, Unnikrisnan, and Hussein 2018)—with the resultant porous space creating a transient negative bone balance that can reduce the stiffness, strength, and fatigue resistance of bone until the site is fully replenished by secondary mineralization, an extensive process that can take upwards of 30 months (Boivin et al., 2009; Plotkin, 2014; Hughes et al., 2017; Mancuso et al., 2022). Conversely, adaptive bone formation, a process of formation modeling, is the independent action of osteoblasts uncoupled from prior osteoclastic bone resorption remodeling that ultimately deposits bone at mechanically advantageous places intended to improve the morphology of the structure (Hughes et al., 2017; Hughes and Bouxsein, 2020). Further, adaptive bone formation, bypassing the resorption period with a median duration of 30–40 days (Eriksen, 2010), is temporally more expedient than is remodeling, foregoing the transient porosity and subsequent increased risk of bone stress injury.

Reports from *in vivo* loading models dating back to the 1960s have established the ability to capture these complex physiologic pathways in animal species (Frost, 2003). Such studies have shown improvement in bone mineral density, trabecular microstructure, and bone geometry in as little as 4 weeks (Mustafy et al., 2019). In another, after 8 weeks of 10, 45 cm freefall drops, bone volume fraction, trabecular and cortical thickness improved in rat ulnas, with greater adaptation resulting from impact than from calcium supplementation (Welch et al., 2008). A study performed over 16 weeks subjected rats to 360 load cycles per day, resulting in significant increases in biomechanical resilience, specifically ultimate force and energy to failure, occurring despite only modest gains in areal bone mineral density (aBMD) and bone mineral content (BMC) (Robling and Turner, 2009). These results have provided the scope in which exercise interventions in human research commonly range in duration from just 4–12 weeks (Linke, Gallo, and Norman, 2011; Hecksteden et al., 2018; Ashton et al., 2020). However, due to rodent cortical bone lacking well-developed haversian remodeling activity (Bentolila et al., 1998)—and the majority of animal models designed for compact bone—resorption data from animal models is limited (Robling and Turner, 2009). Capturing modeling and remodeling in response to exercise in humans is even more challenging, with recent improvements as higher resolution technologies become available.

High-Resolution Peripheral Quantitative Computed Tomography (HR-pQCT) has the potential to capture small and subtle changes in human bone (Mancuso and Troy, 2020). HR-pQCT allows for the assessment of bone compartments by assessing three-dimensional volumetric bone mineral density (vBMD) and structural measurements of total, trabecular and cortical bone due to its isotropic voxel size of 61 μm (XtremeCT II) or 82 μm (XtremeCT) (Whittier et al., 2020). In addition to differentiating between skeletal compartments, advanced HR-pQCT software allows for microfinite element analysis (FEA), capable of estimating the mechanical competence of bone (Boutroy et al., 2007). The ability to discern small changes in bone microarchitecture over a short time has the unequivocal potential to determine intervention efficacy within healthy populations and preemptively identify those at unsuspecting risk of injury or fracture. Additionally, early detection of even modest changes in bone microstructure and morphology contribute to large changes in bone strength, even *in lieu* of changes to bone density or mass (Turner and Robling, 2003; Bass, Eser, and Daly, 2005). Therefore, the purpose of this review is to synthesize existing training-induced HR-pQCT data in three distinct, healthy, adult populations, identify similarities and contrasts with prior modalities and cumulatively interpret results within the scope of bone functional adaptation.

## 2 Selection of studies

Eligibility was confirmed by full-text screening of papers written in English that utilized the HR-pQCT within a healthy, non-diseased, adult (>18 years) population. Reviews, case-reports, conference proceedings and editorials were excluded. Only studies that included an exercise or training intervention (i.e., non-observational) without the use of pharmacological drugs or nutritional supplementation were included. Consequently, 10 studies were included and were further stratified into 3 groups: 1) Military interventions without supplementation (4 studies), 2) Non-osteoporotic, healthy older adults interventions (3 studies), and 2) General population interventions (3 studies). All studies featured here are also presented in Tables 1, 2.

**TABLE 1:** Mean percent differences in total volumetric bone mineral density and trabecular thickness by group.

Study, date	Sex	Age	Length of intervention	Tibial site	Group	Graph icon	Tt.vBMD (mean % difference)		Tb.Th (mean % difference)
Military training									
Hughes et al. (2018)	F	21.5	8 weeks	Metaphysis	Intervention	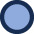	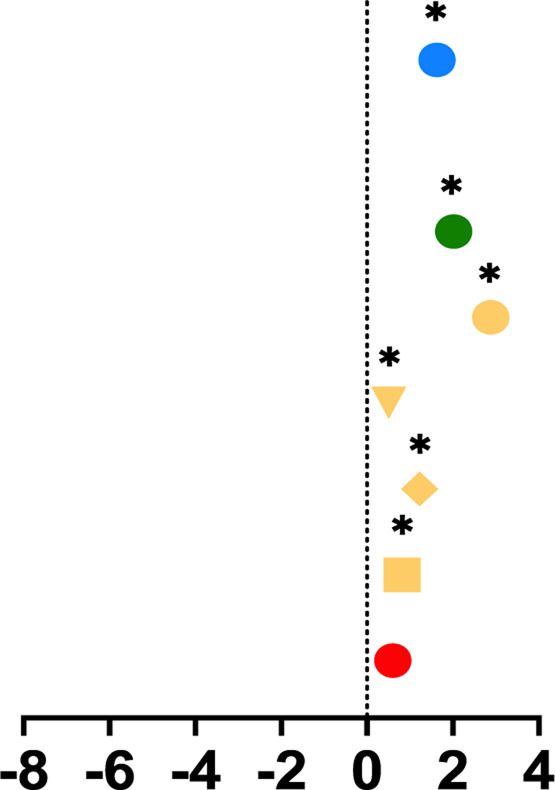		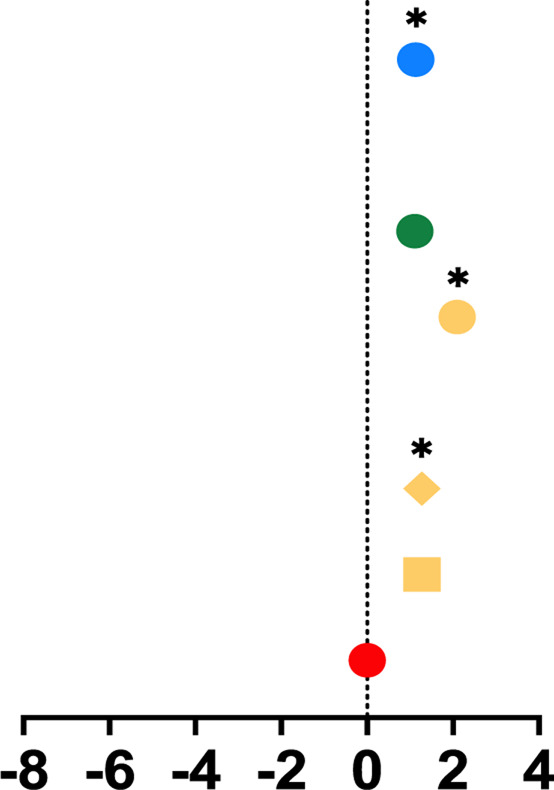
O’Leary et al. (2019)	M	21	13 weeks	Metaphysis	Intervention	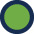			
O’Leary et al. (2021)	F	24	14 weeks	Metaphysis	Intervention	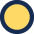			
			14 weeks	Diaphysis	Intervention	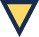			
			28 weeks	Metaphysis	Intervention	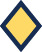			
			44 weeks	Metaphysis	Intervention	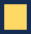			
			44 weeks	Diaphysis	Intervention	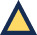			
O’Leary et al. (2019)	F	32	61 days	Metaphysis	Intervention	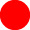			
Non-osteoporotic older adults									
Sundh et al. (2018)	F	55.5	3 months	Ultradistal	Control	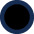	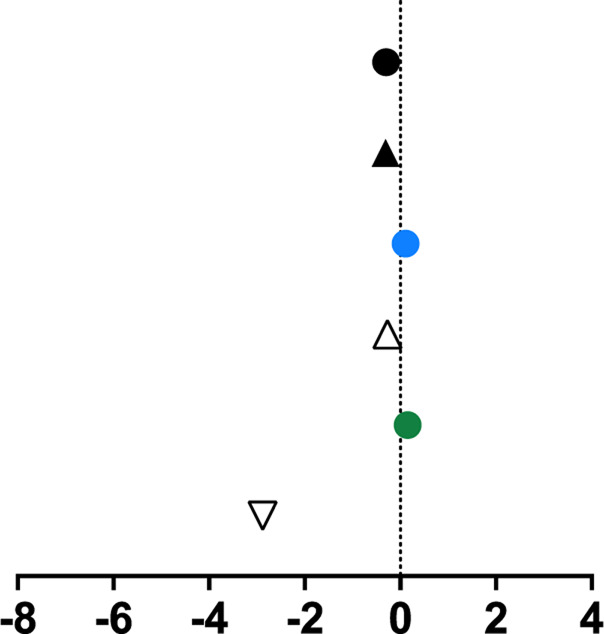		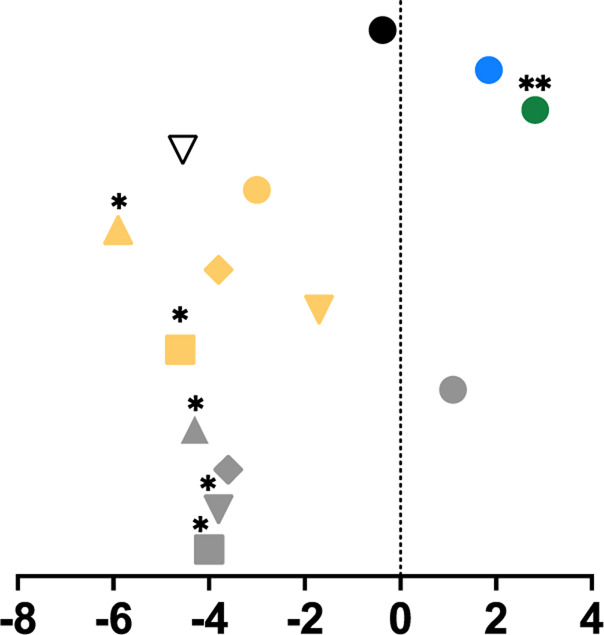
			3 months	Diaphysis	Control	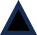			
			3 months	Ultradistal	Exercise	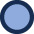			
			3 months	Diaphysis	Control	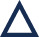			
Pinho et al. (2020)	F	66.9	20 weeks	Metaphysis	Experimental	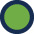			
		65	20 weeks	Metaphysis	Control	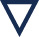			
Du et al. (2021)	F	63	6 months	Metaphysis	Exercise	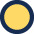			
			6 months	Anterior	Exercise	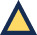			
			6 months	Lateral	Exercise	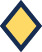			
			6 months	Posterior	Exercise	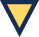			
			6 months	Medial	Exercise	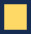			
			6 months	Metaphysis	Control	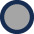			
			6 months	Anterior	Control	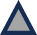			
			6 months	Lateral	Control	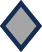			
			6 months	Posterior	Control	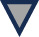			
			6 months	Medial	Control	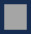			
General population									
Belavy (2011)	M	33.1	30 days	Metaphysis	Control	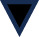	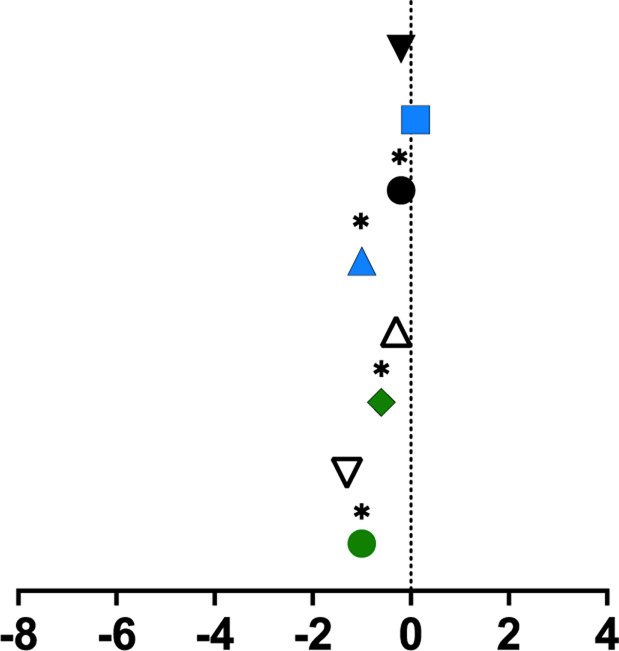		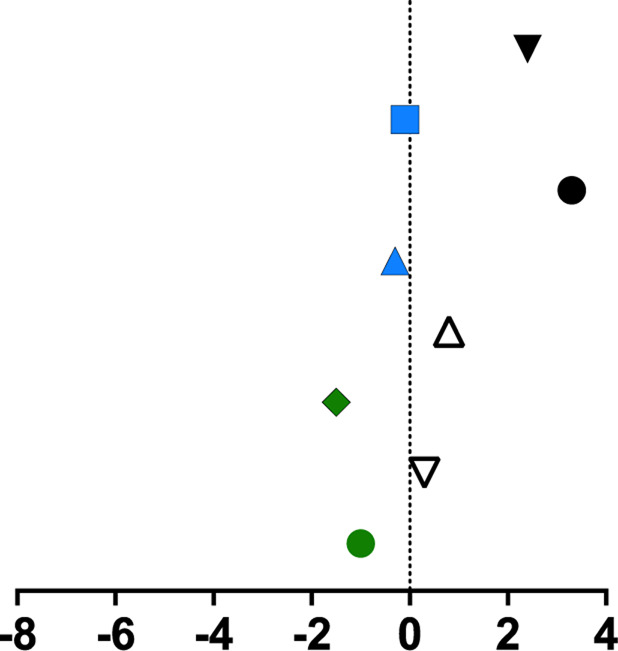
			30 days	Metaphysis	Resistive exercise	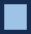			
			59 days	Metaphysis	Control	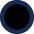			
			59 days	Metaphysis	Resistive exercise	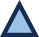			
Armbrecht et al. (2011)	F	34.4	15 days	Metaphysis	Control	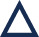			
		32.8	15 days	Metaphysis	Exercise	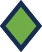			
		34.4	43 days	Metaphysis	Control	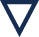			
		32.8	43 days	Metaphysis	Exercise	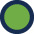			

M, male; F, female; anterior, lateral, posterior, medial, region of whole cross-section; * = significantly different from baseline, ** = significantly different from control group. Troy et al. (2020) excluded from table as only the radius was measured. Exercise or intervention groups denoted in color (yellow, blue, green, red) while control groups denoted in white, black, grey.

**TABLE 2:** Percent changes in bone parameters by group.

Participants	Scanner/Sites(s) assessed	Training duration	Percent changes in bone parameters					Study
			Densitometric parameters (i.e., Tt,Tb,Ct.vBMD, TMD, inn and meta)	Trabecular microarchitecture (i.e., Tb.Th, N, Sp, BV/TV)	Cortical microarchitecture (i.e., Ct.Po, Ct.Po.Dm, Ct.Po.V,Ct.Th, Ct.BV)	Geometric parameters (i.e., Tt, Tb. Ct.Ar, Tb.BS, Ct.Pm)	Mechanical strength (i.e., S, F.ult, VM, strain magnitude)	
Military training without supplementation								
Female Officer Cadets, n = 51, (24 ± 2 years)	Xtreme CT II; 4% and 30% tibia scanned at weeks 1, 14, 28, and 44.	44 weeks of British Army Officer training	4% site: Week 44: +0.3% No change in Ct.vBMD. 30% site: Week 14: -0.3% No change in Tt vBMD	4% site: Week 44: −3.1 to +3.5% 30% site: No significant changes.	4% site: Week 44: +4.0% in Ct.Th 30% site: +0.5% in Ct.Pm.	4% site: Week 44: 0.4 to +4.8% 30% site: Week 44: +0.5%	4% site: No significant changes in S or F.ult. 30% site: Week 44: +2.5% in F.ult.	O’Leary et al. (2021)
Male recruits, n = 43, (21 ± 3 years)	Xtreme CT I; standard tibia of dominant (DL) and non-dominant leg (NDL)	13 weeks of British Army Basic Training	DL:+0.6–1.7% NDL: +0.9–2.02%	DL:+1.05% in BV/TV. NDL: +2.1% in BV/TV. No significant changes in N, Th, Sp in either leg.	DL:+3.1% in Ct.Th. NDL: +3.8% in Ct.Th. No significant change in Ct.Po in either leg.	−0.4 to +2.7% in Tb. And Ct.Ar in the D and NDL. No change in Ct. Pm at either leg.	No significant changes in S or F.ult at either leg but pre and post averages were significantly different from DL at the same timepoint.	O'Leary et al. (2019a)
Female recruits n = 91 (21.5 ± 3.3 years)	Xtreme CT II; 4% and 30% tibia	8 weeks of US Army Basic Combat Training	4% site: −0.38 to +2.01% 30% site: −0.72- (−0.71)%	4% site: −1.09 to +1.87% 30% site: No significant changes	4% site: +0.98% 30% site: No significant changes	4% site: Not measured at 4% site 30% site: No significant changes	4% site: +2.41–2.45% 30% site: +0.21–0.77%	Hughes et al. (2018)
Healthy service women, n = 6 (32 ± 3 years)	Xtreme CT II, 4% and 30% tibia, performed 39 days before expedition and 15 days after.	61-day all-female trans-Antarctic traverse	4% site: No change in Tt., Tb, or Ct.vBMD 30% site: No change in Ct.vBMD	4% site: No significant change 30% site: Parameters not assessed.	No change in Th, Po, Po.Dm at 4 or 30% site	No change in Tb.Ar, Ct.Ar. or Ct.Pm at 4 or 30% site.	No change in F.ult or S at 4 or 30% site	O'Leary et al. (2019b)
Non-osteoporotic, healthy older adults								
Women, experimental group n = 21 (66.9 ± 4.2 years) and control group n = 17 (65 ± 3.4 years)	Xtreme CT I, distal tibia	20 weeks of high impact exercises and power training	E = −0.8% C = −3.0% in Tb.BMD. No significant change in Ct.BMD in either group.	E = +2.8% C = −4.5% in Tb.Th. No significant change in N, BV/TV or Sp in either group.	No significant changes in either group.	No significant change in Tb.BS.	No significant changes in either group.	Pinho et al. (2020)
Healthy, postmenopausal Women, n = 10 (63 ± 4 years)	Xtreme CT I, bilateral standard tibia analyzed by region (anterior, lateral, posterior, medial, global)	6-month unilateral hopping protocol compared to randomly assigned exercise leg (EL) to control leg (CL) of same subject	Parameters not assessed.	Anterior region EL = −5.9 to +6.1% CL = −4.3 to +2.6% Medial region EL = −4.6% CL = −4.0% Globally EL = +4.4% CL = −0.7% No significant changes in lateral or posterior region.	Parameters not assessed.	Parameters not assessed.	No significant changes in S in either leg or region.	Du et al. (2021)
Healthy but inactive postmenopausal women n = 20 (55.5 ± 2.3 years)	Xtreme CT I, bilateral standard and 14% tibia and analyzed by region (anterior, lateral, posterior, medial, global)	3-month daily one-legged jumps with increased frequency compared to control leg	No significant changes at standard or 14% site. Tb.vBMD not measured.	No significant changes at standard or 14% site nor in any region.	No significant changes at standard or 14% site nor in any region.	No significant changes at standard or 14% site nor in any region.	Parameters not measured.	Sundh et al. (2018)
General population								
Healthy women n = 102 (28 ± 6 years) assigned to control group or one of two exercise arms: 1) low and high strain magnitude 2) low and high strain rate	Xtreme CT I, bilateral standard radius at baseline, 3, 6, 9 and 12 months.	12 mo. of 100 cycles of axial force 4x weekly	Control: No significant changes at any timepoint. Low: 3 mo.: 0.39% 9 mo.: 0.44%–2.59% High: 3 mo.: 0.29%–2.78% 9 mo.: -0.26%	No significant changes at any timepoint in any group in Tb.N or Th	No significant changes at any timepoint in any group except for the high rate group at 3 mo.: +3.74% in Ct.Th	Parameters not assessed.	Parameters not assessed.	Troy et al. (2020)
Healthy men n = 24 assigned to bed rest with no exercise (33.1 ± 7.8 years) or bed rest plus restrictive exercise (31.1 ± 5.1 years)	Xtreme CT I; standard tibia and standard radius performed at 3 days prior to bedrest, days 30 and 59 of bedrest, and 3, 15, 30, 180, 360, and 720 days after bedrest.	60 days of head down tilt bed rest with no exercise (controls), or with resistive exercise (RE) during bedrest. Control group did not partake in countermeasures during bedrest, the RE group performed resistive exercises 3 days a week for the duration of bedrest.	Radius: CON: Significant changes seen at post-720 days with a 0.9% increase in CtvBMD and a −1.5% decrease in Meta RE: Significant change seen at post-720 with a +0.8% increase in CtvBMD Tibia: CON: Significant decreases were seen in density measures from 59 to post-720 ranging from −3.0% to −0.4% RE: Significant changes were seen in density measures from 30 to post-30 ranging from −1.1 to +0.3%	Radius: No significant changes in any group. Tibia: CON: Significant changes seen at 59, and post-90 to post-720 ranging from −4.2 to +4.9% RE: Significant decreases ranging from −3.7 to (−3.2%) seen at post-90	Radius: CON: Significant changes seen at post-720 with +0.2% increase RE: Significant changes seen at post-15, post-90, post-360, and post-720 with an increase of +1.5 to 2.4% in Ct.Th Tibia: CON: Significant changes seen at 59 and post-3 to post-90 ranging from −2.9 to +0.3% RE: Significant changes seen 59, post-3, post-15, and post-30 ranging from −2.1 to +0.3%	Radius**:** CON: Significant change seen at post-720 with a −0.4% decrease in TbAr but an 1.4% increase in CtAr RE: Significant change seen at post-90 and post-720 with a decrease of −0.7 to (−0.4)% in TbAr but a +1.2 to 2.4% increase in CtAr Tibia**:** CON: Significant changes seen at 59, post-3, post-15, post-30, and post-90 with decreases in CtAr from −1.9% to −2.5% and a 0.4% increase in TbAr RE: Significant changes seen at 59, post-3, post-15, post-30, post-360 and post-720 with decreases in CtAr from −2.0 to (−1.5)% and changes in TbAr from −0.3 to +0.3%	Parameters not assessed.	Belavý (2011)
Female volunteers n = 24 were assigned to one of two groups: 1) control [CON] (34.4 ± 3.8 years), 2) regular muscular exercise [EXE] (32.8 ± 3.4 years)	Xtreme CT I, standard distal radius and distal tibia performed 9 days prior to bedrest, days 15 and 43 of bed rest and 3,90, 180 and 360 days after bedrest.	Experimental protocol lasted 100 days: 20 days of baseline measures, 60 days of 6° head- down tilt bed rest, and 20 days of post-bedrest recovery. Control group did not partake in countermeasures during bedrest, EXE group performed resistance training every 2–3 days and aerobic exercise on a supine treadmill every 3–4 days.	Radius: CON: Significant changes seen only at post-3 days with a −0.5% decrease in Tb.vBMD EXE: Significant changes seen at 15, post-3, post-180 ranging from −1.8- (−0.6)% No significant change in any group at any timepoint in Meta or Ct.vBMD. Tibia: CON: Significant changes seen at post-3, post-90, post-360 days ranging from −4.9 to (−0.6)% EXE: Significant changes seen at 15, 43, post-3, post-90, post-180, post-360 ranging from −3.0 to (−0.1)%	Radius: No significant change in any group at any timepoint in Tb.N or Th. CON: Significant changes seen at post-180 and post- 360 days in Tb.Sp ranging from +2.6 to 3.1% EXE: Significant changes seen at 15, post-3 and post-18 days in BV/TV ranging from −0.14 to (−0.10)% Tibia: CON: Significant changes seen at post-3, post-360 days ranging from −3.1 to +3.7% EXE: Significant changes seen at 15, 43, post-3, post-90, post-180, post-360 ranging from −0.38 to +2.1%	Radius: No significant change in any group at any timepoint in Ct.Th. Tibia: CON: Significant changes seen at 43, post-3, post-90, post-180 ranging from −2.0 to (−0.6)% EXE: Significant changes seen at 43, post-3, post-90, ranging from −0.9 to +0.1%	Parameters not assessed.	Parameters not assessed.	Armbrecht et al. (2011)

## 3 Military interventions

Perhaps the best evidence we have to support the ability of the HR-pQCT to detect short-term yet meaningful changes in skeletal microarchitecture lies in studies of basic military training (BCT), as these 8–44 weeks programs represents a brief period of often unaccustomed physical activity. Prior to the advent of the HR-pQCT, Peripheral Quantitative Computed Tomography (pQCT) studies demonstrated modest anabolic changes in bone density, geometry and strength in response to short-term (<10 weeks) exercise interventions in male cadets (Izard et al., 2016) and in density, content and cortical microarchitecture in female cadets (Gaffney-Stomberg et al., 2014), though others, demonstrated no changes (Baker et al., 2022). However, pQCT studies have also consistently reported that certain geometric phenotypes (smaller/narrower bone, lower cross-sectional area) predispose military cadets to bone stress injury (Jepsen et al., 2011; Wentz et al., 2011; Davey et al., 2015; O’Leary et al., 2021), though some results have been inconsistent in women(Moran et al., 2008; Cosman et al., 2013). pQCT holds some advantages over HR-pQCT, specifically capturing midshaft and proximal sites and the ability to measure muscle cross sectional area. However, pQCT suffers from partial volume artifacts and lacks the necessary resolution (170 μm) to examine bone microarchitecture (Nishiyama and Shane, 2013). While limited, current HR-pQCT evidence from field training environments demonstrates that short periods of military training (<13 weeks) can elicit favorable adaptations in the tibia, including increased density, size and strength in both women (Hughes et al., 2018) and men (O’Leary et al., 2019a).

In just 8 weeks of Army BCT, 91 female cadets (21.5 ± 3.3 years) experienced a significant increase of approximately 2% in total and trabecular vBMD and trabecular BV/TV at the tibial metaphysis, as well as 1.13%–1.21% increases in trabecular number and thickness (Hughes et al., 2018). Changes however were less robust at the cortical compartment as cortical thickness increased by almost 1% while cortical vBMD and TMD decreased in the tibial metaphysis and diaphysis (Hughes et al., 2018). Stiffness and failure load increased at both sites, conferring much larger magnitude changes at the metaphysis (2.41%–2.45% versus 0.21%–0.77% at diaphysis), though both changes were significant. In 13 weeks of British Army BCT, 43 young men (21 ± 3 years) experienced a significant increase in total (1.7%–2.02%), trabecular (1.3%–1.9%) and cortical density (0.56%–0.9%) as well as Tb BV/TV (1.05%–2.1%), cortical area (2.7%) and cortical thickness (3.1%–3.8%) at the distal tibia. Trabecular area (−0.4%) significantly declined while trabecular number, thickness, spacing, cortical perimeter and porosity did not significantly change (O’Leary et al., 2019a). Longer training (44 weeks) in female Officer cadets demonstrated greater changes in trabecular and cortical microarchitecture at the metaphysis than did shorter training, demonstrating a magnitude of change 1.5–3 fold higher than any previous military study, with the largest changes occurring at the metaphysis in trabecular vBMD (3%), trabecular number (3.5%), cortical thickness (4%) and cortical area (4.8%) (O’Leary et al., 2021).

BCT environments elicit mild energy deficits (Adler et al., 2015; O’Leary et al., 2021). Alternatively, longitudinal assessment of bone microarchitecture under conditions of severe physiological stress are limited. One such example included six healthy British servicewomen (32 ± 3 years) traversing the Antarctic, scanned via HR-pQCT approximately 1 month before the expedition and 2 weeks afterwards. As a result, there were no changes at the tibial metaphysis or diaphysis. The authors reported significant declines in axial aBMD ranging from −2.6% to −5% (O’Leary et al., 2019b) with concurrent preservation of the appendicular skeleton and bone biomarkers indicative of remodeling (O’Leary et al., 2019). Bone resorption, specifically, has been shown to increase with military training (Evans et al., 2008; Lutz et al., 2012; Hughes et al., 2014). HR-pQCT, by neither capturing discernable gains in vBMD nor improvements in microarchitecture, indicated that intense physical activity conducted under multiple stressors (i.e., prolonged severe exercise stress and reduced energy availability) does not favor anabolic formation modeling in women. Aforementioned changes in vBMD and microarchitecture over a short period of time are highly indicative of bone formation modeling, or *de novo* bone formation (Hughes et al., 2017; Hughes and Bouxsein, 2020) on existing surfaces, without prior resorption. Training-induced decreases in cortical vBMD at both sites also occurred, suggestive of potentially concurrent intracortical targeted remodeling. This suggests that bones can adapt in structure and strength to even brief periods of heightened physical activity in healthy, young adult populations. As many of the military trainees are beyond the age of peak height velocity and residual bone deposition due to growth, these HR-pQCT in military trainees offer evidence that even the mature young adult skeleton retains mechanosensitivity.

### 3.1 General population interventions

Prior pQCT studies have demonstrated density-centric skeletal adaptations in response to short-term training within general population cohorts. For example, trabecular density increased by approximately 1.34% at the distal tibia in 20-year-old women following just 8 weeks of aerobic (i.e., running based) training, along with a modest improvement in aBMD (Lester et al., 2009). Similarly, trabecular density increased by approximately 0.8%–1.2% in the distal tibia following 13 weeks of combined aerobic and periodized resistance training in young women(R. K. Evans et al., 2012). However, bone properties are only partially explained by bone density as measured by pQCT, as we know that bone microarchitecture is a key determinant of bone’s mechanical competence (Jepsen et al., 2011; Manske et al., 2015) fragility (Burr et al., 1996; Carbonare and Giannini, 2004) and can discriminate those with and without fracture (Legrand et al., 2000; Davey et al., 2015). To date, few studies utilizing the HR-pQCT have been performed in general, non-specialized populations. For example, 102 healthy women (28 ± 6 years) were scanned bilaterally at the standard radius at baseline, 3, 6, 9 and 12 months while undergoing 12 months of 100 cycles of axial force, four times per week. The low stimulus group observed significant increases at 3 (0.39%) and 9 months (0.44%–2.59%) in total and trabecular BMD. The high stimulus group similarly reported increases at 3 (0.29%–2.78%) but a decline at 9 (−0.26%) months in total and trabecular density, indicating formation modeling with increases in cortical thickness at 3 months and perhaps concurrent targeted remodeling specifically with decreases in inner-trabecular vBMD at 9 months (Troy et al., 2020).

Exercise loads the skeleton, in contrast, bedrest—a frequent analog for spaceflight—unloads bone. Bone loss due to situations of disuse typically occurs in bone mass and density in weight-bearing regions, namely, the legs, hip and pelvis (LeBlanc et al., 2000). Twenty four healthy men (31.1 ± 5.1 years) and 24 women (32.8 ± 3.4 years) underwent 60 days of bed rest (60 days) with or without resistive exercise (EXE) 3x per week that targeted the lower extremity (i.e., bilateral squats, back and heel raises) (Armbrecht et al., 2011; Belavý, 2011). Women in the EXE group performed resistance training every 2–3 days and aerobic exercise on a supine treadmill every 3–4 days (Armbrecht et al., 2011). Distal tibia and radial scans were performed prior to, during, and 1–2 years post-bedrest. At the radius, men experienced a slight but significant increase in Ct.vBMD (0.8%), Ct.Ar (1.2%–2.4%) and Ct.Th (1.5%–2.4%) while Tb.Ar progressively worsened (Belavý, 2011). There was no change in trabecular microarchitecture except for Tb.1/N.SD—a measure of inhomogeneity—which decreased significantly by −3.7% 90 days into recovery. At the tibia, density parameters (−1.1% to 0.3%), geometric (−2.0% to 0.3%) and cortical microarchitecture (−2.1% to 0.3%) only modestly changed with the most pronounced changes occurring in trabecular microarchitecture (−3.7% to −3.2%) (Belavý, 2011). There was no evidence of impact from exercise on any skeletal parameter at either site in women (Armbrecht et al., 2011). In men, exercise blunted significant declines in trabecular microarchitecture compared with the control group (Belavý, 2011). However, this occurred only in the tibia, with little to no benefit seen at the radius. Conclusively, exercise during prolonged bedrest was insufficient in counteracting the consequences of disuse. As expected, HR-pQCT parameters at the distal tibia showed greater changes than the distal radius, consistent with weight-bearing sites demonstrating greater sensitivity to inactivity than non-weight-bearing sites. These studies also provided evidence of sex-specific differences in response to disuse as women demonstrated significantly greater losses than did men. A limitation however is that, in disuse-mediated remodeling, deficits preferentially occur at the endocortical region in diaphyseal bone (Hughes and Bouxsein, 2020). Therefore, as only distal sites were studied in both instances, it is plausible that greater skeletal deficits went unnoticed.

### 3.2 Healthy, non-osteoporotic older adults

Exercise of sufficient load and impact (Petit, Hughes, and Warpeha, 2008) can mitigate bone loss that occurs with aging. While the current diagnosis of osteoporosis is based on areal bone mineral density (aBMD; g/cm^2^) values, it is been shown that the aBMD in most older adults who experience a fracture is outside of the T-score denoting osteoporotic range (<−2.5) (Mikolajewicz et al., 2020). Additionally, DXA’s measurement of two-dimensional areal bone mineral density (aBMD) is commonly employed as a surrogate measure of bone strength but lacks the resolution to distinguish between cortical and trabecular bone (Harding and Beck, 2017). However, we know from DXA studies that even short-duration exercise interventions can promote bone formation in an aging skeleton. For example, 16 weeks of resistance training in 59–61 year old men resulted in a 2.8%–3.8% increase in femoral neck BMD compared with no changes seen in non-exercising controls (Menkes et al., 1993; Ryan et al., 1994). While aging women tended to need combined training and longer interventions (25 weeks–6 months) to ascertain skeletal benefit (Harding and Beck, 2017), 25 weeks of combined weight lifting and agility training in women aged 75–85 years significantly increased cortical BMD as measured by pQCT at the proximal tibia and radius by 0.5%–1.9% (Liu-Ambrose et al., 2004). With large benefits discernable in older populations with lower resolution technologies, we can anticipate important future findings related to microarchitecture in those that employ HR-pQCT. Few HR-pQCT studies consist of a healthy, non-osteoporotic aging population, none of which include men. Twenty healthy but inactive postmenopausal women (55.5 ± 2.3 years) undergoing 3-months of daily one-legged jumps observed no significant changes in densitometric or geometric parameters, trabecular or cortical microarchitecture or mechanical strength at the distal or ultradistal tibial (Sundh et al., 2018). Similarly, 20 weeks (5 months) of high-impact exercises in 38 osteopenic women aged 60–70 years, elicited only modest responses including a −0.8% reduction in Tb.vBMD and 2.8% increase in Tb.Th at the tibial metaphysis (Pinho et al., 2020). A 6-month unilateral hopping protocol significantly increased trabecular microarchitecture, specifically at the global—whole cross section—and anterior region with a 4.4% increase globally and 6.1% increase in BV/TV anteriorly, but no significant change in mechanical strength at the tibial metaphysis (Du et al., 2021). While more diverse research is still needed, HR-pQCT studies confirm that favorable anabolic changes to the distal tibia in <6 months are possible in a senescent skeleton.

## 4 Discussion

Following short-term training (8–13 weeks) robust changes are observed during military training with sex-specific differences in bone adaptation. Military initial level training involves a drastic and sudden increase in both volume and frequency of weight bearing activities such as unaccustomed load carriage and military drill, during which the lower extremities experience intense loading (O’Leary et al., 2021). Greater sensitivity to heightened mechanical loading consistently occurred distally, despite animal studies demonstrating that long bone adaptations in response to mechanical loading tends to occur at the midshaft and more proximally (Warden et al., 2004; Wallace et al., 2007). This discrepancy could be the result of limited scan breadth on HR-pQCT lending to best capturing only the distal appendicular skeleton (Lieberman et al., 2003; Pearson and Lieberman, 2004). However, the second generation HR-pQCT allows for expansion of the diaphyseal region to include up to 66% of total tibial length. Although to date, no training-specific studies have utilized this methodology, future longitudinal capture at this site will allow for further elucidation of cortical bone, muscle and fat in response to exercise or training (Whittier et al., 2020). Longer training periods in female Officer cadets demonstrated greater changes in trabecular and cortical microarchitecture at the metaphysis than did shorter training, a magnitude of change 1.5-3 fold higher than any prior military study (O’Leary et al., 2021). Maintenance of whole body aBMD in conjunction with these high magnitude adaptations implies that unaccustomed mechanical loading was protective against periodic low energy availability(Gifford et al., 2021) despite significant changes in total body, fat and lean mass(O’Leary et al., 2021). Further research is needed to further elucidate factors promoting versus preventing adaptation, from which we’ll discover new risk factors (i.e., energy availability, psychological stress, sleep, NSAIDs) (Hughes et al., 2022) for bone stress injury, stress fracture and bone fragility.

Within the general population, healthy adult women undergoing 12 months of compressive loading of the forearm showed significant increases in BMC associated with strain ranges experienced during activities of daily living (Troy et al., 2020). This finding conflicts with the previously held belief that extremely high strain magnitudes were necessary to elicit an adaptive response, specifically in women (Martyn-St James and Carroll, 2010). In the absence of loading, men demonstrated a pattern of bone recovery after disuse most pronounced at the distal tibia with recovery of cortical bone (namely, Ct.Ar and Ct.Th) beginning as early as 3 days after bedrest (Belavý, 2011). Women, however, demonstrated the greatest loss in Ct.Th at the distal tibia, despite additional protein supplementation (Armbrecht et al., 2011). These findings demonstrate sex-specific differences in skeletal recovery following disuse, such that men require substantially less time than do women for recovery of bone structure and density (Armbrecht et al., 2011).

Drawing sex-specific conclusions in aging populations remains difficult, as few HR-pQCT studies exist with healthy, aging populations and those that do, include exclusively women. However, existing research demonstrates that bone mechanosensitivity remains viable in women well into their 6th decade of life with changes favoring the distal trabecular compartment. Cumulatively, these results demonstrate that age may not be the decisive factor in whether or not an adaptive response occurs, but instead the length of the intervention (>6 months), type of stimulus (multidirectional, high impact) and granularity of the imaging technique (regional analysis). There remains work to be done to establish the effectiveness of exercise not just in preventing bone loss but encouraging anabolism into old age. The exercise-specific characteristics and environmental considerations that will promote these processes have yet to be fully elucidated and captured with HR-pQCT technology.

Introduction of the HR-pQCT has provided researchers the ability to investigate bone microarchitecture *in vivo* and quantify densitometric, morphological and geometric adaptation in response to exercise and training exposure. While pharmaceutical interventions have shown efficacy, they are often contraindicated until late in life. Non-pharmacological, non-invasive training intervention studies provide insight into the optimal exercise program to enhance bone strength and reduce susceptibility to bone injury, stress and osteoporotic fracture. Ultimately, the utilization of highly sensitive bone imaging modalities like the HR-pQCT maximizes the ability to detect even modest change that may, in turn, manifest into substantial effects over longer periods of time. Promising horizons are ahead for the role of exercise to promote anabolism and bone strength across the lifespan.

## Figures and Tables

**Table:** 

**Abbreviation**	Meaning	Typical Units
**aBMD**	Areal bone mineral density	g/cm^2^
**Ct.Ar**	Cortical area	cm^2^
**Ct.Pm**	Cortical perimeter	mm
**Ct.Po**	Cortical porosity	%
**Ct.Po.Dm**	Cortical Pore Diameter	µm
**Ct.Th**	Cortical thickness	mm
**Ct.TMD**	Cortical tissue mineral density	mg HA/cm^3^
**Ct.vBMD**	Cortical volumetric bone mineral density	mg HA/cm^3^
**DXA**	Dual energy X-ray Absorptiometry	
**F.ult**	Failure load	kN
**HR-pQCT**	High-resolution peripheral quantitative computed tomography	
**pQCT**	Peripheral quantitative computed tomography	
**S**	Whole bone stiffness	kN/mm
**Tb.Ar**	Trabecular area	cm^2^
**Tb. BV/TV**	Trabecular bone volume/total volume	%
**Tb.BS**	Trabecular surface area	mm^2^
**Tb.N**	Trabecular number	1/mm
**Tb.Sp**	Trabecular spacing	mm
**Tb.1/N.SD**	Std. deviation of trabecular spacing (trabecular heterogeneity)	mm
**Tb.Th**	Trabecular thickness	mm
**Tb.vBMD**	Trabecular volumetric bone mineral density	mg HA/cm^3^
**Tt.BMD**	Total volumetric density	mg HA/cm^3^
**vBMD**	Volumetric bone mineral density	mg HA/cm^3^
**VM**	Von Mises stresses	MPa
